# Revascularization of Chronic Total Occlusions vs. Planned Complex Percutaneous Coronary Intervention: Long-Term Outcomes and Mortality

**DOI:** 10.3390/jcm14030758

**Published:** 2025-01-24

**Authors:** Marcel Almendarez, Alberto Alperi, Isaac Pascual, Rut Alvarez-Velasco, Rebeca Lorca, Daniel Hernández-Vaquero, José Luis Betanzos, Juan Francisco Ortiz de Zarate, Raul Ptaszynski, Paula Antuña, Luis Arboine, Pablo Avanzas

**Affiliations:** 1 Heart Area, Hospital Universitario Central de Asturias, Avenida de Roma S/N, 33011 Oviedo, Spain; marcel.almendarez@gmail.com (M.A.); alberto.alperi.garcia@hotmail.com (A.A.); rutalvarez3@gmail.com (R.A.-V.); lorcarebeca@gmail.com (R.L.); betanzos42@hotmail.com (J.L.B.); r.ptaszynski95@gmail.com (R.P.); paulantu@hotmail.com (P.A.); avanzaspablo@uniovi.es (P.A.); 2 Health Research Institute of Asturias, Avenida de Roma S/N, 33011 Oviedo, Spain; 3 Faculty of Medicine, University of Oviedo, 33006 Oviedo, Spain; 4 Interventional Cardiology Department, Unidad Médica de Alta Especialidad, Hospital de Cardiología 34, Monterrey 64360, Mexico; luisarboine1986@gmail.com; 5 Centro de Investigación en Red de Enfermedades Cardiovasculares (CIBERCV), 28029 Madrid, Spain

**Keywords:** percutaneous coronary intervention, chronic total occlusion, all-cause death, myocardial infarction, target vessel revascularization

## Abstract

**Introduction:** The number of chronic total occlusion (CTO) revascularization procedures has continuously increased, obtaining better results in recent years. However, there are few data regarding long-term outcomes and no comparisons to planned complex non-CTO percutaneous coronary intervention (PCI). **Methods:** We included all patients undergoing planned complex PCI. Our main objective was to compare a combined endpoint of all-cause death, myocardial infarction, and target vessel revascularization at the long-term follow-up of CTO PCI versus planned complex non-CTO PCI. We compared the groups using multivariable Cox regression and performed a propensity score matching analysis to control the baseline characteristics. We repeated the analysis for the separate components of the primary endpoint. **Results:** From January 2018 to June 2023, 1394 complex coronary PCIs were performed at our center. After excluding 393 non-planned cases, 201 CTO PCIs and 800 non-CTO PCIs were included. The mean follow-up was 2.5 ± 1.5 years. The composite endpoint occurred in 23 (11.6%) CTO PCIs and 219 (28.2%) planned non-CTO PCIs. The multivariable Cox regression using the CTO group as the reference showed a lower risk for the primary outcome (HR: 0.59; 95% CI 0.37–0.95; *p* = 0.031). After matching, a total of 195 adequately balanced pairs were obtained. The CTO group presented a lower risk for the primary combined outcome (HR: 0.46; 95% CI 0.27–0.76; *p* = 0.003). **Conclusions:** In patients undergoing planned complex PCI, those in the CTO group presented a reduced risk of all-cause death, myocardial infarction, and target vessel revascularization at the end of the follow-up.

## 1. Introduction

Cardiovascular disease is the leading cause of worldwide morbidity and mortality, and exponential growth is expected in the following years, with studies projecting a rise in the prevalence of elderly patients, obesity, and metabolic disorders. A higher burden of these risk factors will result in increased numbers of atherosclerotic coronary disease. Furthermore, the effectiveness of revascularization may be limited by restenosis of previously treated vessels and may require subsequent interventions [1]. Of all the cases diagnosed with coronary artery disease, up to twenty percent will present with a chronic total occlusion (CTO).

Revascularization of CTO requires meticulous procedural planning, highly experienced interventionalists, and adequate patient selection to ensure better results. The main indications are to treat refractory angina, subjects with large areas of documented ischemia in the region of the occluded vessel, and to improve quality of life whenever optimal medical treatment (OMT) is insufficient to control symptoms [2,3].

Operators may be discouraged from CTO revascularization due to low success rates, a higher number of complications, and a controversial overall benefit with contradictory results regarding symptom control compared to OMT [4]. However, due to the immense prevalence of coronary artery disease, the overall number of cases with a clear indication for intervention is growing. Furthermore, increased operator and center experience, along with continuous advancements in recanalization algorithms and the development of new medical devices, will produce a yearly increase in the volume of CTO revascularization [5,6]. Therefore, a detailed comparison of its long-term results and complications is warranted.

Most of the available literature is focused on success rates, technical improvements, and benefits of CTO percutaneous coronary intervention (PCI) compared to OMT without comparing to non-CTO PCI [7,8]. Only a handful of registries have compared CTO revascularization to non-CTO PCI, and they include simple and complex interventions for all-comers with short follow-up periods. These groups are hardly comparable to each other, considering the technical difficulty and the preparation required for a CTO PCI [9,10]. Only one study has compared CTO PCI versus complex non-CTO PCI with a significant follow-up period. However, in this analysis, patients with stable CAD and acute coronary syndrome (ACS) were included [11]. Comparing patients in an acute scenario to a staged procedure, like most CTO PCI cases, may act as a confounder. For this reason, we designed this study to compare the results of CTO PCI to planned complex non-CTO PCI.

## 2. Materials and Methods

This retrospective study included all patients who underwent PCI at our center from January 2018 to June 2023. We analyzed planned complex PCI according to a modified version of previously published criteria, including CTO PCI, three vessels treated, ≥three lesions treated, left main PCI with one or more stents, bifurcation PCI with two stents excluding left main, aortocoronary bypass graft intervention, severe calcification requiring atherectomy (including laser or lithotripsy), and use of mechanical circulatory support in nonemergency cases [12,13,14,15]. CTO was defined as a 100% stenosis with thrombolysis in myocardial infarction (TIMI) 0 flow for over three months diagnosed in a previous angiography or estimated according to the clinical history and angiographic appearance [16].

Procedures were performed electively by the same team of CTO/complex PCI operators. The X-ray system was an Artis Zee (Siemens AG, Forcheim, Germany) with cine angiography in a single plane at a frame rate of 15/s with a 20 cm zoom. Cases with heavy calcification assessed by fluoroscopy or intracoronary imaging (intravascular ultrasound, optical coherence tomography) were managed according to the operator with modified balloons and ablation therapies such as rotational atherectomy and/or intravascular lithotripsy.

CTO PCI was indicated in cases of refractory angina, evidence of ischemia, or both. For the rest of the complex non-CTO PCIs, unplanned procedures such as ST-elevation myocardial infarction (STEMI), patients in cardiogenic shock, and non-STEMI with immediate revascularization after the initial angiography were excluded unless discussed with the CTO/complex PCI team and a strategy was defined beforehand. Ad hoc procedures were discouraged.

Procedural success was defined as achieving a TIMI 2 flow or greater in all ≥2.5 mm distal branches and residual stenosis of <30% at the end of the procedure, and the absence of in-hospital major adverse cardiovascular events. Staged procedures to facilitate complete revascularization or CTO modification with a scheduled subsequent PCI were not considered major adverse cardiovascular events (MACE). Only the last planned intervention was included in the analysis [16].

The baseline characteristics, procedural, hospitalization, and discharge data were obtained from a prospectively collected PCI database. Follow-up data were obtained from the electronic clinical history, phone interviews, and outpatient visits for patients needing more information. All patients signed an informed consent form. Standardized endpoints and causes of death were defined according to the Academic Research Consortium (ARC)-2 consensus document and the CTO-ARC consensus recommendations [16,17].

### 2.1. Endpoints

Our primary endpoint was a composite of all-cause death, any MI, or clinically driven target vessel revascularization (TVR) at the long-term follow-up. Our secondary endpoints were the separate components of the composite endpoint.

### 2.2. Statistical Analyses

Categorical variables are shown as numbers (percentages) and compared with the chi-squared test and, in the case of several categories, with the ANOVA test; quantitative variables were expressed as mean ± standard deviation or median (interquartile range) and compared with the Student’s *t*-test and Wilcoxon’s rank test, respectively.

We performed a multivariable Cox regression to determine the factors that predicted mortality. The variables introduced were defined using a backward stepwise regression with a cutoff *p*-value < 0.10. Associations were expressed as the hazard ratio (HR) with a 95% confidence interval (95% CI). The model’s predictive capacity was evaluated with Harrel’s C test and survival using the Kaplan–Meier curves. The differences were statistically significant, with a *p*-value of <0.05.

Finally, to reduce the variability that may exist in the baseline characteristics of CTO PCI versus non-CTO PCI, we performed a propensity score (PS) matching analysis. We included all baseline characteristics and calculated mortality with a Cox regression. We matched using the nearest neighbor method without replacement with a caliper of 0.05. We compared baseline characteristics with the mean standardized difference (MSD). An absolute value of >0.10 was considered significant. We graphed the effect after matching using balancing diagnostics and visual graphics [18,19,20]. If a satisfactory balance among groups was obtained, survival curves using the Kaplan–Meier method were performed and compared with the log-rank test. Hazard ratios were estimated with a Cox regression in the matched sample using CTO as the reference population. Statistical analysis was performed with STATA 16 IC (StataCorp, College Station, TX, USA).

## 3. Results

### 3.1. Baseline Characteristics

From January 2018 until June 2023, 5704 PCIs were performed at our center. Following the complex coronary criteria, 1394 cases were selected. After excluding 312 STEMIs, 26 patients in cardiogenic shock, and 55 non-STEMIs with immediate revascularization without discussing the case with the coronary complex team, 1001 patients were included for analysis. There were 201 CTO PCIs (20.1%) and 800 (79.9%) planned complex non-CTO PCIs.

Most cases were male, totaling 775 (77.4%), and the mean age was 71.1 years. CTO patients were younger (67.5 vs. 72 years, *p* < 0.001), presented fewer cases of chronic kidney disease (CKD) (10.5% vs. 21.5%, *p* < 0.001), and had a higher rate of previous coronary artery bypass graft (CABG) (4% vs. 9.6%, *p* = 0.01). The rest of the baseline characteristics can be consulted in Table 1.

### 3.2. Procedure and Hospitalization

The mean J-CTO score for the CTO group was 2.1 ± 0.9, and 19.4% of the cases were performed with a retrograde approach. Procedural data were worse for the CTO group, with higher contrast volume, doses of radiation, and fluoroscopy time. Most CTO cases (78%) required two vascular access devices, with 59.2% through the femoral artery (59.2%). The most common complexity feature for CTO was a total stent length > 60 mm in 40.3% of the cases; however, for non-COT PCI, left main PCI was significantly higher, with 296 cases (37% vs. 3.5%, *p* < 0.001). The complex non-CTO group showed higher rates of ≥3 lesions (13% vs. 7%, *p* = 0.021), left main intervention with two stents (10.3% vs. 3.5%, *p* = 0.002), and lithotripsy (11.5% vs. 3%, *p* = 0.046). Procedural success was achieved in 80.1% and 97.1% (*p* < 0.001) for CTO vs. non-CTO PCI, respectively. Table 2 contains the rest of the procedural data and complexity features.

Both groups had similar complications during the procedure except for perforations requiring intervention or pericardial drainage in 3% of the CTOs compared to 1% in the non-CTO group (*p* = 0.032). During the hospitalization, there were no significant differences in major bleeding, vascular complications, contrast-induced nephropathy (CIN), and in-hospital death (1.5% vs. 2.8%, *p* = 0.572). Complications can be consulted in Table 3.

### 3.3. Follow-Up

The mean follow-up was 2.5 ± 1.5 years. The composite endpoint of all-cause death, any MI, and clinically driven TVR occurred in 23 (11.6%) CTO PCIs and 219 (28.2%) non-CTO PCIs. There was a total of 12 deaths (6.1%) vs. 134 (17.3%), 5 (2.5%) MI vs. 100 (12.9%), and 10 (5.1%) TVR vs. 64 (8.2%) subsequently for the CTO group vs. the non-CTO group. Details can be consulted in Table 3.

**Figure 1:**
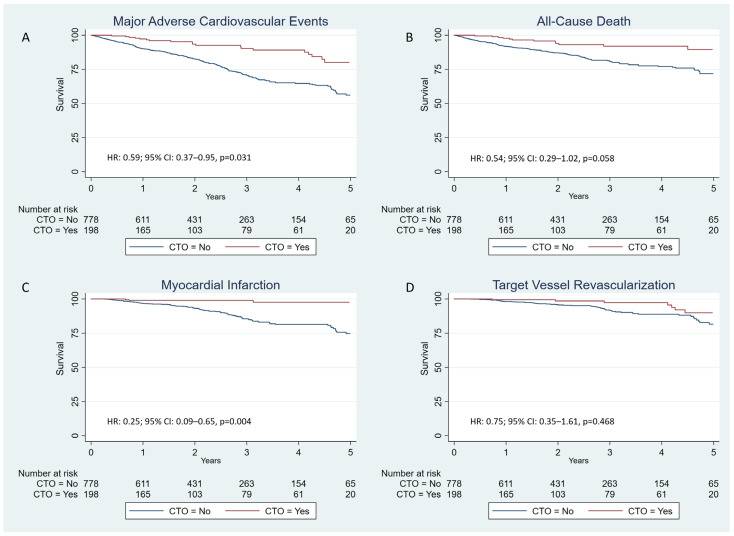
Kaplan–Meier cumulative incidence estimates: (**A**) combined primary endpoint (all-cause death, myocardial infarction, target vessel revascularization); (**B**) all-cause death; (**C**) myocardial infarction; (**D**) target vessel revascularization.

We performed a multivariable Cox regression for the main outcome and components separately. The hazard ratio using the CTO group as the reference for the main outcome was 0.59; 95% CI 0.37–0.95; *p* = 0.031 (Figure 1A). The predictors of the main event were age, diabetes, number of diseased vessels, bifurcations requiring two stents, and LVEF (Table 4). The reference group tended to have a lower risk of death, with the Kaplan–Meier curves separating from the beginning of the follow-up but without reaching statistical significance (HR: 0.54; 95% CI: 0.29–1.02; *p* = 0.058) (Figure 1B). In the case of any MI, the Kaplan–Meier survival curves show that CTO procedures were associated with lower risk, with an HR of 0.25 and a 95% CI of 0.09–0.65; *p* = 0.004 (Figure 1C). There were no differences regarding TVR (HR: 0.75; 95% CI: 0.35–1.61; *p* = 0.468) (Figure 1D). Details for the multivariable analysis for the secondary endpoints can be consulted in Supplementary Table S1.

### 3.4. Matched Sample

**Figure 2:**
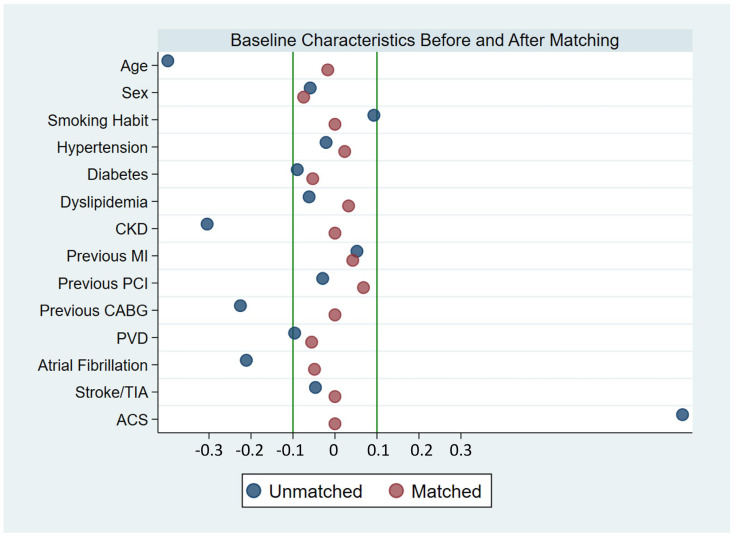
Balancing diagnostics showing variables before (blue circles) and after (red circles) propensity score matching. Mean standardized differences between −0.1 and 0.1 imply good balance.

Analyzing the MSD, the following baseline characteristics were not adequately balanced: age, previous CABG, CKD, peripheral vascular disease, atrial fibrillation, and acute coronary syndrome at admission (Table 1). We performed PS matching to overcome this drawback, obtaining 195 correctly balanced pairs (Supplementary Table S2, Figure 2). The matched sample had no significant differences regarding procedural complications, including coronary perforation. The same occurred for complications during hospitalization with similar rates of bleeding, stroke/TIA, CIN, and cardiovascular death (Supplementary Table S3).

**Figure 3:**
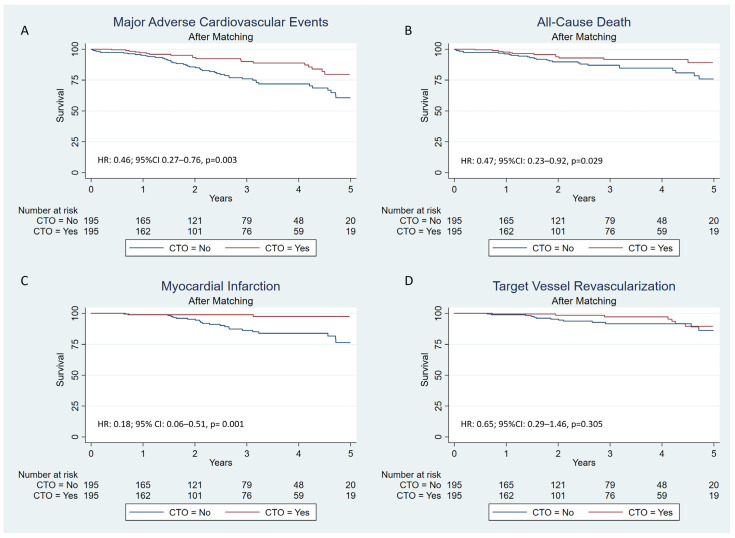
Kaplan–Meier cumulative incidence estimates in the matched cohort: (**A**) combined primary endpoint (all-cause death, myocardial infarction, target vessel revascularization); (**B**) all-cause death; (**C**) myocardial infarction; (**D**) target vessel revascularization.

The mean follow-up was 2.7 ± 1.6 years. Compared to the non-CTO PCI group, patients from the CTO group presented a lower risk for the primary combined outcome (HR: 0.46; 95% CI 0.27–0.76; *p* = 0.003) (Figure 3A), all-cause death (HR: 0.47; 95% CI: 0.23–0.92; *p* = 0.029) (Figure 3B), and any MI (HR: 0.18; 95% CI: 0.06–0.51; *p* = 0.001) (Figure 3C) at the end of the follow-up period. CTO PCI was not an independent predictor for TVR, with similar rates among groups (HR: 0.65; 95% CI: 0.29–1.46; *p* = 0.305) (Figure 3D) (Supplementary Table S4).

## 4. Discussion

**Figure 4:**
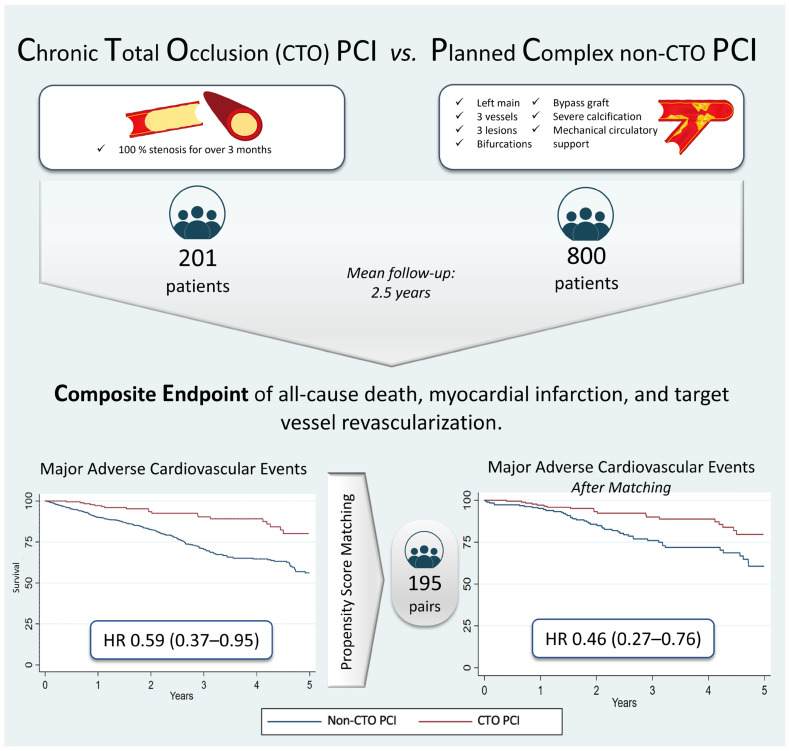
After 2.5 years of follow-up, chronic total occlusion intervention presented a lower risk of the combined endpoint of all-cause death, myocardial infarction, and clinically driven target vessel revascularization compared to planned complex non-occlusive intervention. A propensity score matching analysis obtaining 195 pairs confirmed these findings.

The main finding of our study is that after 2.5 years of follow-up, CTO PCI was not an independent predictor for a combined endpoint comprising all-cause death, any MI, and clinically driven TVR compared to planned complex non-CTO PCI. A similar result was obtained for the individual components of the primary endpoint except for TVR. These findings were confirmed in a multivariable and PS matching analysis (Figure 4).

Considering the frequency of CTOs found in patients with CAD undergoing angiography, a large volume of patients with angina, despite OMT, are expected to benefit from CTO recanalization. This is a continuously growing procedure with improved success rates in recent years due to increased operator experience and advances in the specific material for the intervention [21,22]. It is paramount to determine the long-term outcomes of CTO PCI and predictors for worse clinical outcomes that allow adequate patient selection to improve the success rate and reduce overall complications. Furthermore, it is essential to determine how they compare to a better-established technique like complex non-CTO PCI, unlike other studies focusing on procedural aspects and immediate technical success.

The benefit of CTO PCI over medical therapy has been questioned, with inconclusive results among different randomized trials. The EXPLORE trial randomized 304 patients after a STEMI for early PCI of a CTO vs. conservative treatment. The primary endpoint was LVEF and diastolic volume and showed no differences among groups (44.1 ± 12.2% vs. 44.8 ± 11.9%, respectively; *p* = 0.60) [23]. The DECISION-CTO trial randomized 834 patients to a CTO PCI (*n* = 317) or no CTO PCI (*n* = 398) strategy. There were no significant differences in the primary combined endpoint (22.3% vs. 22.4%; HR: 1.03; 95% CI, 0.77 to 1.37; *p* = 0.86), with questionable benefits regarding quality of life [7]. Nevertheless, these studies were not able to assess hard clinical endpoints; therefore, the overall benefit of CTO PCI remains disputed. Furthermore, repeated revascularization and clinical endpoints compared to non-occlusive PCI were unexplored, making this study more relevant.

Only a small number of studies have compared CTO to non-CTO PCI. A landmark study by Brilakis et al., with data from the National Cardiovascular Landmark registry, analyzed in-hospital results of 22,365 CTO vs. 594,510 non-CTO PCI. Like our study, the overall contrast volume and fluoroscopy time were greater among CTO procedures. They showed a low overall success with 59% vs. 96% (*p* < 0.001), yet experienced operators with >10 cases and centers with >30 cases per year improved to over 75% success, a value similar to our study’s 80.1% success rate. The number of CTO cases per operator in this study was around 40 per year, concurring with their results. The MACE rates were higher for the CTO group (1.6% vs. 0.8, *p* < 0.001). Like the success rate, the MACE rate was reduced to 1% in higher-volume centers [9]. The main drawback of their analysis was that there was no follow-up time, and they compared CTO PCI to all PCIs, with no distinction of complexity.

A similar, more recent study using data from the National Inpatient Sample Database with over 10 million cases undergoing PCI and 259,574 cases of CTO PCI showed a mortality rate of 3.17% for CTO and 2.57% for the rest of PCI (odds ratio 1.07; 95% CI 1.02–1.13), a relatively small difference despite comparing complex and simple PCI cases. Our in-hospital mortality rate was lower, 1.5% for CTO PCI and 2.8% for non-CTO PCI. The perforation rate was higher for CTO cases (OR 6.01; 95% CI 5.25–6.89). The perforation rate in our series was 3% for CTO cases and only 1% for the rest. These data must be taken cautiously since the database is retrospective with no reference to the center/operator volume and no follow-up period [10].

Our findings reinforce Azzalini et al.’s findings, which compared long-term outcomes of CTO to complex non-CTO PCI. A key difference was that they included unstable patients in cardiogenic shock and patients with STEMI. In our analysis, we excluded this group of patients since it is an entirely different scenario and could influence the results of CTO PCI. However, there were many similarities. The overall procedural metrics were worse (i.e., contrast volume and fluoroscopy time), and there was a higher incidence of perforation and similar rates for in-hospital MACE. At the end of the follow-up period, the combined endpoint of death, target lesion revascularization, and MI was similar for both groups, and CTO PCI was not a predictor for the main event in the multivariable analysis (10.1% vs. 9.9%, *p* = 0.91) [11].

Our results provide complementary data since we analyzed planned complex coronary interventions, arguably the most similar scenario to a CTO recanalization. To control for possible confounders, we performed a multivariable analysis identifying previous PCI, the number of diseased vessels, two-stent bifurcation, and the LVEF as independent predictors for the combined outcome. These results were driven fundamentally by a significantly higher rate of MI in the non-CTO group during the follow-up. Likewise, all-cause death showed a tendency to fewer cases in the CTO group without reaching statistical significance. CTO was not a predictor of clinically driven TVR.

A further sensitivity analysis with a propensity score matched 195 pairs with correctly balanced baseline characteristics. Interestingly, after matching, non-CTO PCI was an independent predictor of all-cause death and MI at the long-term follow-up. TVR remained similar for both groups.

This study has some limitations. First, it presents the inherent biases of observational and retrospective studies. Second, it lacks the systematic use of intravascular images and pre-procedural computerized tomography, which have shown benefits in complex PCI [24]. The results warrant further studies with a randomized design comparing the long-term outcomes of CTO PCI. However, the study presents several strengths that intend to minimize its limitations. This is a large cohort of complex PCI with a long-term follow-up and meticulous evaluation of each case. We only included staged procedures to eliminate the variability of acute coronary syndrome. Furthermore, we obtained well-balanced groups by controlling possible confounders via multivariable and PS matching analysis.

## 5. Conclusions

In conclusion, in this single-center cohort of patients undergoing complex PCI, those in the CTO group exhibited a reduced risk of MACE at the end of the follow-up. After balancing with a PS-matched analysis, CTO patients presented a lower risk for all-cause death and MI. There were no differences regarding clinically driven TVR.

## Figures and Tables

**Table 1:** Baseline characteristics.

	Overall (*n* = 1001)	CTO (*n* = 201)	Complex Non-CTO (*n* = 800)	*p*	MSD
Age (years)	71.1 ± 11.2	67.5 ± 11.1	72 ± 11	<0.001	−0.4
Sex (men)	775 (77.4%)	159 (79.1%)	616 (77.0%)	0.524	−0.06
Hypertension	739 (73.8%)	147 (73.1%)	592 (74.0%)	0.803	−0.02
Diabetes	389 (38.9%)	71 (35.3%)	318 (39.8%)	0.250	−0.09
Dyslipidemia	667 (66.6%)	129 (64.2%)	538 (67.3%)	0.409	−0.06
Smoking habit Smokers Ex-smokers	196 (19.6%) 334 (33.4%)	47 (23.4%) 67 (33.3%)	149 (18.6%) 267 (33.4%)	0.458	0.09
Previous MI	365 (36.5%)	78 (38.8%)	287 (35.9%)	0.440	0.05
Previous PCI	291 (29.1%)	57 (28.4%)	234 (29.3%)	0.803	−0.03
Previous CABG	85 (8.5%)	8 (4.0%)	77 (9.6%)	0.010	−0.23
CKD	193 (19.3%)	21 (10.5%)	172 (21.5%)	<0.001	−0.3
Stroke/TIA	85 (8.5%)	15 (7.5%)	7 (8.8%)	0.558	−0.05
PVD	173 (17.3%)	29 (14.4%)	144 (18%)	0.231	−0.1
Atrial fibrillation	134 (13.4%)	16 (8%)	118 (15%)	0.115	−0.21
ACS - No - UA/NSTEMI	457 (45.6%) 544 (54.4%)	153 (76.1%) 45 (23.9%)	304 (38%) 496 (62%)	<0.001	0.8

Variables are shown as mean ± standard deviation for quantitative variables and number (%) for categorical variables. Abbreviations: ACS: acute coronary syndrome; CABG: coronary artery bypass grafting; CKD: chronic kidney disease; CTO: chronic total occlusion; MI: myocardial infarction; NSTEMI: non-ST elevation myocardial infarction; PVD: peripheral vascular disease; SD: Mean standardized difference; TIA: transient ischemic attack; UA: unstable angina.

**Table 2:** Angiographic characteristics.

Variable	CTO (*n* = 201)	Complex Non-CTO (*n* = 800)	*p*
Number of diseased vessels	1.2 ± 0.5	1.3 ± 0.6	0.001
1 disease vessel	144	375	<0.001
2 disease vessels	40	278	<0.001
3 disease vessels	17	157	<0.001
Number of vessels treated	1.3 ± 0.7	1.7 ± 0.8	<0.001
1 vessel treated	166	543	0.006
2 vessels treated	31	214	<0.001
3 vessels treated	4	42	<0.001
Vessel treated			
Left main	14 (7%)	379 (47.4%)	<0.001
Left anterior descending	82 (40.8%)	473 (59.1%)	<0.001
Left circumflex	45 (22.4%)	232 (29%)	0.062
Right coronary artery	104 (51.7%)	244 (30.5%)	<0.001
Severe calcification	81 (40.3%)	287 (35.9%)	0.243
Severe tortuosity	17 (8.5%)	58 (7.3%)	0.567
Total stent length (mm)	49.1 ± 30	65.9 ± 29	<0.001
Number of stents implanted	2 ± 1.4	2.2 ± 1.2	0.276
Contrast volume (mL)	257 ± 129	180 ± 79	<0.001
Fluoroscopy time (min)	42.1 ± 31.5	25.8 ± 97.9	0.021
Radiation dose (mGy)	4728 ± 3196	2825 ± 3562	<0.001
Intra-aortic balloon pump	12 (6%)	65 (8.15%)	0.314
Femoral access	119 (59.2%)	268 (33.5%)	<0.001
Double access	149 (78%)	21 (3%)	<0.001
Procedural success	161 (80.1%)	777 (97.1%)	<0.001
Complex PCI characteristics			
Total stent length > 60 mm	81 (40.3%)	273 (34.1%)	0.101
3-vessel PCI	6 (3%)	37 (4.6%)	0.311
≥3 lesions	14 (7%)	104 (13%)	0.021
Left main intervention	7 (3.5%)	296 (37%)	<0.001
Left main intervention with two stents	7 (3.5%)	82 (10.3%)	<0.002
Two-stent bifurcation	5 (2.5%)	38 (4.8%)	0.157
Bypass graft intervention	1 (0.5%)	26 (3.3%)	0.031
Atherectomy	25 (12.4%)	144 (18%)	0.060
Lithotripsy	6 (3%)	92 (11.5%)	0.046
Mechanical circulatory support	5 (2.5%)	35 (4.4%)	0.223

Variables are shown as mean ± standard deviation for quantitative variables and number (%) for categorical variables. Abbreviations: CTO: chronic total occlusion; PCI: percutaneous coronary intervention.

**Table 3:** Complications.

Variable	CTO (*n* = 201)	Complex Non-CTO (*n* = 800)	*p*
Intraprocedural			
Perforation	6 (3%)	8 (1%)	0.032
Side-branch occlusion	2 (1%)	8 (1%)	1
Ventricular arrhythmias	3 (1.5%)	7 (0.9%)	0.427
Orotracheal intubation	2 (1%)	8 (1%)	1
Cardiac arrest	2 (1%)	2 (0.3%)	0.135
Intraprocedural death	0	1 (0.13%)	0.615
Failed vascular closure device	2 (1%)	9 (1.1%)	0.874
Hospitalization			
Vascular complication	10 (5%)	28 (3.5%)	0.328
Bleeding	10 (5%)	47 (5.9%)	0.623
BARC classification - 1 - 2 - 3A - 3B - 5A - 5B	- 1 (0.5%) - 5 (2.5%) - 1 (0.5%) - 3 (1.5%) - 0 - 0	- 4 (0.5%) - 21 (2.6%) - 15 (1.9%) - 5 (0.63%) - 1 (0.1%) - 1 (0.1%)	0.568
CIN	18 (9%)	82 (10.3%)	0.584
Stroke/TIA	2 (1%)	10 (1.3%)	0.767
In-hospital death	3 (1.5%)	22 (2.8%)	0.307
LVEF	52.6 ± 10.8	53.1 ± 10.8	0.572
Follow-up			
Composite of all-cause death, MI, or TVR	23 (11.6%)	219 (28.2%)	<0.001
All-cause death	12 (6.1%)	134 (17.3%)	<0.001
MI	5 (2.5%)	100 (12.9%)	<0.001
Clinically driven TVR	10 (5.1%)	64 (8.2%)	0.132

Variables are represented as mean ± standard deviation for quantitative variables and number (%) for categorical variables. Abbreviations: BARC: bleeding academic research consortium; CIN: contrast-induced nephropathy; CTO: chronic total occlusion; MI: myocardial infarction; TIA: transient ischemic attack; TVR: target vessel revascularization.

**Table 4:** Univariate and multivariable analysis of the primary endpoint.

Variable	Univariate			Multivariable		
	HR	95% CI	*p*	HR	95% CI	*p*
CTO	0.37	0.24–0.57	<0.001	0.59	0.37–0.95	0.031
Age	1.06	1.04–1.08	<0.001	1.02	1.01–1.04	0.001
Sex	1.13	0.78–1.63	0.525	1.03	0.75–1.42	0.853
Diabetes	1.98	1.43–2.75	<0.001	1.52	1.15–2	0.003
Previous PCI	1.53	1.09–2.15	0.014	1.42	1.05–1.92	0.022
Number of diseased vessels	1.35	1.11–1.65	0.002	1.22	1.03–1.45	0.023
2-stent bifurcation	1.75	1.16–2.65	0.008	1.54	1.05–2.27	0.027
LVEF	0.96	0.95–0.98	<0.001	0.98	0.97–1	0.076
Smoking habit	0.76	0.54–1.05	0.1			
Hypertension	1.38	0.93–2.04	0.111			
Dyslipidemia	0.98	0.69–1.38	0.91			
CKD	2.47	1.75–3.51	<0.001	1.31	0.94–1.84	0.111
Stroke/TIA	1.68	0.99–2.82	0.052	0.79	0.47–1.32	0.36
PVD	1.76	1.19–2.64	0.005	1.39	0.97–2.02	0.075
Atrial fibrillation	1.6	1.06–2.4	0.024	1.09	0.75–1.58	0.636
ACS	1.16	0.96–1.40	0.115			
Left main disease	1.52	1.09–2.14	0.014	1.16	0.86–1.56	0.322
Femoral access	1.52	1.18–1.97	0.001	1.17	0.88–1.55	0.279
Lithotripsy	0.69	033–1.41	0.316			
IABP	2.5	1.54–4.05	<0.001	1.38	0.83–2.26	0.211
Rota-ablation	1.84	1.27–2.6	0.001	1.21	0.86–1.69	0.267
Total stent length	0.99	0.99–1.01	0.378			
CIN	1.35	0.82–2.21	0.234			

Abbreviations: ACS: acute coronary syndrome; CIN: contrast-induced nephropathy; CKD: chronic kidney disease; CTO: chronic total occlusion; IABP: intra-aortic balloon pump; LVEF: left ventricle ejection fraction; NSTEMI: non-ST elevation myocardial infarction; PCI: percutaneous coronary intervention; PVD: peripheral vascular disease; TIA: transient ischemic attack.

## Data Availability

Data will be made available upon request.

## Supplementary Materials

The following supporting information can be downloaded at https://www.mdpi.com/article/10.3390/jcm14030758/s1: Table S1: Multivariable analysis of the secondary endpoints; Table S2: Baseline characteristics after matching; Table S3: Complications after matching.
