# Supplementary material for: Revascularization of Chronic Total Occlusions vs. Planned Complex Percutaneous Coronary Intervention: Long-Term Outcomes and Mortality
Source: J Clin Med. 2025 Jan 24;14(3):758. doi: 10.3390/jcm14030758 (PMC11818758; doi:10.3390/jcm14030758)
Supplement: Supplementary file 1 [file jcm-14-00758-s001.zip › jcm-3399658-supplementary.pdf]

Supplementary Materials:

Supplementary table 1. Multivariable analysis of the secondary endpoints.

Variable	HR	P	95% CI
	Death		
CTO	0.54	0.058	0.29-1.02
Age	1.05	<0.001	1.03-1.08
Diabetes	1.75	0.002	1.22-2.51
LVEF	0.98	0.002	0.96-0.99
	Myocardial infarction		
CTO	0.25	0.004	0.09-0.65
Age	1	0.967	0.99-1.01
PVD	2.98	<0.001	1.75-5.06
Number of diseased vessels	1.35	0.029	1.03-1.78
	Target vessel revascularization		
CTO	0.75	0.468	0.35-1.61
Age	1	0.979	0.99-1
PVD	2.58	0.005	1.33-4.98

Abbreviations. **CTO**: Chronic total occlusion. **LVEF:** Left ventricle ejection fraction. **PVD:** Peripheral vascular disease.

Supplementary table 2. Baseline characteristics after matching.

	**CTO (n = 195)**	**Complex non-CTO (n =195)**	**p**	**SD**
Age (years)	68.8 ± 10.7	68 ± 11.4	0.868	-0.02
Men	156 (80 %)	150 (77.0%)	0.460	-0.07
Hypertension	53 (27.2 %)	55 (28.2%)	0.821	0.02
Diabetes	69 (35.4 %)	74 (38 %)	0.599	-0.05
Dyslipidemia	126 (64.6 %)	123 (63.1%)	0.752	0.03
Smoke Habit				
-Current Smokers	46 (23.6 %)	43 (22.1 %)	0.873	-0.01
-Ex-smokers	64 (32.8 %)	67 (34.4 %)		
Previous MI	73 (37.4 %)	69 (35.4 %)	0.674	0.04
Previous PCI	56 (28.7 %)	50 (25.6 %)	0.495	0.07
Previous CABG	8 (4.1 %)	8 (4.1 %)	1	0
CKD	21 (10.8%)	21 (10.8%)	1	0
Stroke/TIA	15 (7.7%)	15 (7.7%)	1	0
PVD	29 (14.9%)	33 (16.9 %)	0.580	-0.06
Atrial fibrillation	16 (8.2%)	19 (9.7%)	0.595	-0.05

ACS<br>- No<br>- UA/NSTEMI	147 (75.4%)<br>48 (24.6%)	147 (75.4%)<br>48 (24.6%)	1	0

Variables are represented as mean ± standard deviation for quantitative variables and number (%) for categorical variables. Abbreviations. **ACS:** Acute coronary syndrome. **CABG:** Coronary artery bypass grafting. **CKD:** Chronic kidney disease. **CTO**: Chronic total occlusion. **MI**: Myocardial infarction. **NSTEMI:** Non-ST elevation myocardial infarction. **PVD:** Peripheral vascular disease. **TIA:** Transient ischemic attack. **UA:** Unstable angina.

Supplementary table 3. Complications after matching.

Variable	CTO (n = 195)	Complex non-CTO (n= 195)	p
**Intraprocedural**			
Perforation	6 (3.1%)	3 (1.5%)	0.312
Side-branch occlusion	2 (1%)	1 (0.5%)	0.559
Ventricular arrhythmias	3 (1.5%)	2 (1%)	0.652
Orotracheal intubation	2 (1%)	0	0.156
Cardiac arrest	2 (1%)	0	0.156
Intraprocedural death	0	1 (0.5%)	0.317
Failed vascular closure device	2 (1%)	9 (1.1%)	0.562
**Hospitalization**			
Vascular complication	10 (5.1%)	6 (3.1%)	0.307
Bleeding	10 (5.1%)	9 (4.6%)	0.814
BARC classification			
- 1	- 1 (0.5%)	- 0	
- 2	- 5 (2.6%)	- 4 (2.1%)	
- 3A	- 1 (0.5%)	- 2 (1%)	
- 3B	- 3 (1.5%)	- 2 (1.03%)	
- 5A	- 0	- 0	0.812
- 5B	- 0	- 1 (0.1%)	
CI-AKI	18 (9.2%)	15 (7.7%)	0.585
Stroke/AIT	2 (1%)	4 (2.1%)	0.411
In-hospital death	3 (1.5%)	6 (3.1%)	0.312
LVEF	52.5 ±10.9	52.4 ± 11.3	0.963
**Follow-up**			
Composite of all-cause death, MI, or TVR	23 (12%)	49 (25.9%)	<0.001
All-cause Death	12 (6.3%)	27 (14.3%)	0.010
Any myocardial infarction	5 (2.6%)	24 (12.7%)	<0.001

Clinically driven target vessel revascularization	10 (7.9%)	10 (5.2%)	0.282

Variables are represented as mean ± standard deviation for quantitative variables and number (%) for categorical variables. Abbreviations. **BARC:** Bleeding academic research consortium. **CIN:** Contrast-induced nephropathy. **CTO**: Chronic total occlusion. **MI:** Myocardial infarction. **TIA:** Transient ischemic attack. **TVR:** Target vessel revascularization.

Supplementary table 4. Cox regression after matching.

Variable	HR	95% CI	p
Composite of all-cause death, MI, or TVR	0.46	0.27-0.76	0.003
All-cause Death	0.47	0.23-0.92	0.029
